# Supplementary material for: ProDOL: a general method to determine the degree of labeling for staining optimization and molecular counting
Source: Nat Methods. 2024 Aug 8;21(9):1708–15. doi: 10.1038/s41592-024-02376-6 (PMC11399104; doi:10.1038/s41592-024-02376-6)
Supplement: Supplementary file 1 — Supplementary Figs. 1–3, Note 1 and Table 1. [file 41592_2024_2376_MOESM1_ESM.pdf]

nature methods

Supplementary information https://doi.org/10.1038/s41592-024-02376-6

ProDOL: a general method to determine the degree of labeling for staining optimization and molecular counting

In the format provided by the authors and unedited

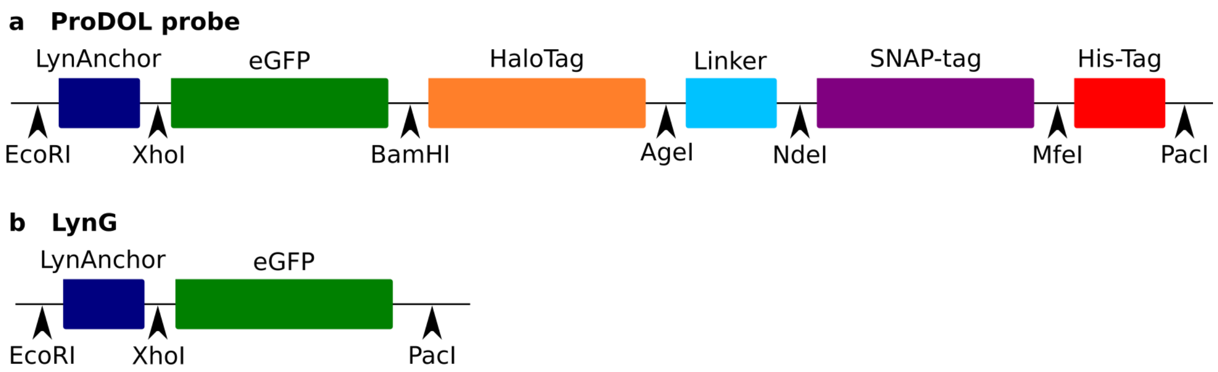

Supplementary Fig. 1: ProDOL and LynG sequence features. a, ProDOL consists of 6 domains (LynAnchor, eGFP, HaloTag, Linker, SNAP-tag, and His-Tag) separated by short (6 bp) unique restriction sites (EcoRI, XhoI, BamHI, AgeI, NdeI, MfeI, and PacI) allowing for easy modifiability of the construct. **b**, Truncated ProDOL probe named LynG (LynAnchor-eGFP) utilised as negative labelling control. For colour coded sequences see below.

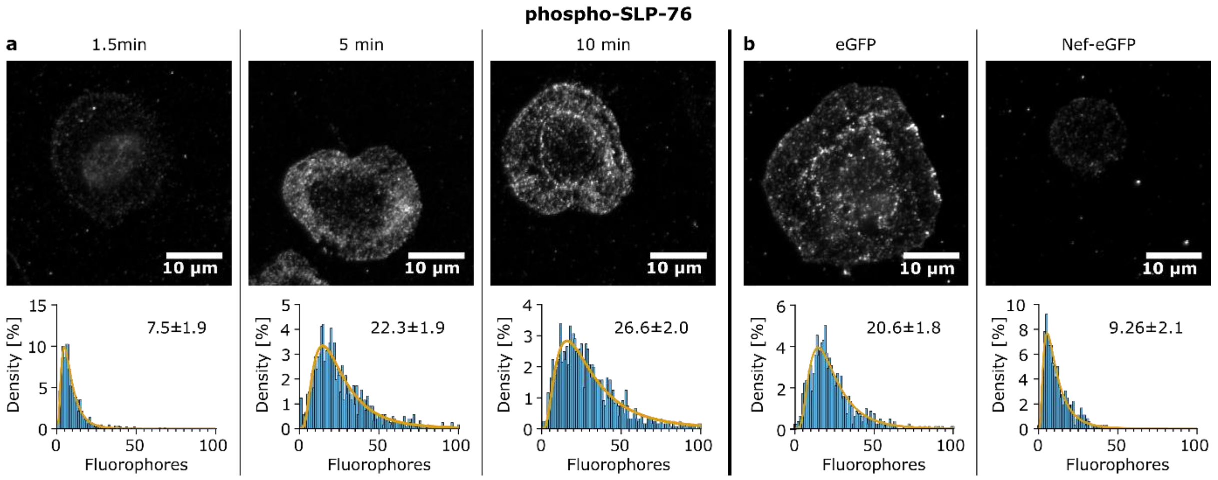

Supplementary Fig. 2: Changes of phosphorylation of SLP-76 with time and effects of the viral protein Nef. a, Jurkat CD4 T cells labelled with anti-pY145-SLP-76 at different times of activation. **b**, Jurkat cells transfected with eGFP or Nef-eGFP labelled with anti-pY145-SLP-76. The bottom panels show histograms of the absolute number of the antibodies per MC (blue) with a log-normal probability function (orange). **a**, Cluster analysis for 1.5 min: n=800 clusters, 5 min: n=1211 clusters, 10 min: n=1206 clusters. **b**, cluster analysis for eGFP: n=822 clusters, Nef-eGFP: n=358 clusters; orange line: best fit of log-normal distribution.

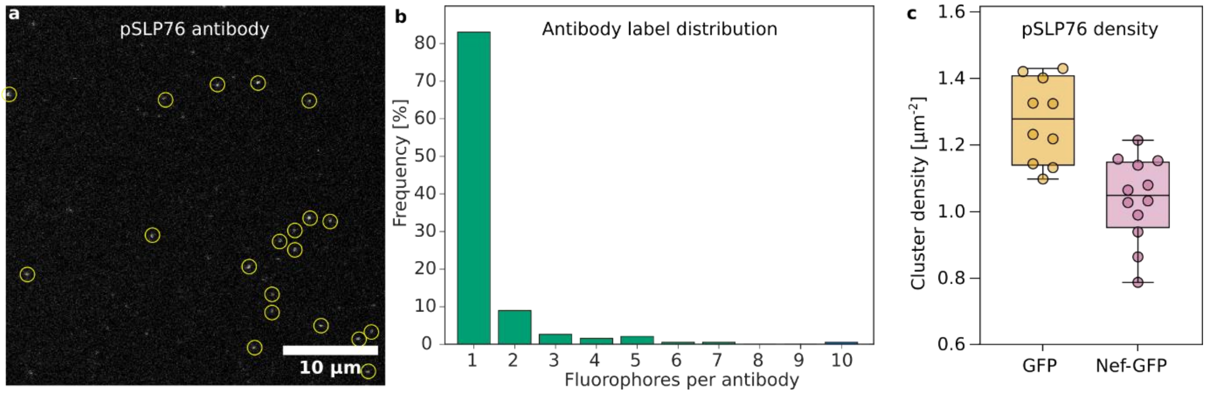

Supplementary Fig. 3: Distribution of dyes per pSLP-76-antibody and pSLP-76 cluster density in cells. **a**, representative microscopic image of immobilised pSLP-76 antibodies on a glass coverslip. **b**, Label distribution of pSLP-76 antibodies determined by CoPS. **c**, Distribution of cluster density of pSLP-76 in Jurkat cells expressing Nef-eGFP and negative control (eGFP). Box plots span the interval from the 25th to the 75th percentile with the median indicated by a horizontal line within the box. Whiskers extend to 1.5× the interquartile range. **b**, n=233 antibodies. **c**, n=10 cells for GFP and n=12 cells for Nef-GFP.

Supplementary Note 1. Phosphorylation of SLP-76 in T cell activation and effects of Nef.

In contrast to copy numbers (Fig. 4a), SLP-76 phosphorylation at tyrosine 145 significantly increased over time from 7.5±1.9 detected fluorophores per MC at 1.5 min to 22.3±1.9 at 5 min ($p<0.0001$), and to 26.6±2.0 at 10 min ($p<0.001$) (Supplementary Fig. 2a). Using the dye to antibody ratio (DOL: 1.27±0.05) determined by CoPS measurements on antibodies (Supplementary Fig. 3a,b), a corrected quantity of labelled phospho-SLP-76 per MC can be determined for 1.5 min: (5.9±1.5), 5 min: (17.6±1.6), and 10 minutes (20.9±1.8) accounting for the total population of both SLP-76 and SLP-76-HaloTag. This means that the increase in phosphorylation, and not protein numbers, is the likely responsible for downstream signalling.

Consistent with copy numbers (Fig. 4b), the density of phospho-SLP-76 containing MCs (Supplementary Fig. 3c) and the amount of phospho-SLP-76 per MC was also significantly reduced in presence of Nef (eGFP: 9.26±2.1; Nef-eGFP: 20.6±1.8, $p<0.0001$; DOL corrected: eGFP: 7.3±1.7; Nef-eGFP: 16.2±1.6) (Supplementary Fig. 2b). Notably, the presence of Nef did not affect the proportion of SLP-76 phosphorylation per MC (Nef-eGFP: 45±11% vs eGFP: 50±13% - $358 \leq N \leq 1348$) ($p=0.0620$). Nef thus disrupts SLP76 function at the level of MC recruitment but does not affect the activation of MC resident SLP76.

Supplementary Information

Table S1: DNA sequences of ProDOL and LynG probes. LynAnchor, eGFP, HaloTag, Linker, SNAP-tag, His-tag.

Construct	Sequence
**ProDOL**	ATGGGATGTATCAAGAGTAAGCGTAAGGATAATCTCAATGACGACGAGCTCGAGACC<br>ATGGTGAGCAAGGGCGAGGAGCTGTTCACCGGGGTGGTGCCCATCCTGGTCGAGCT<br>GGACGGCGACGTAAACGGCCACAAGTTCAGCGTGTCCGGCGAGGGCGAGGGCGAT<br>GCCACCTACGGCAAGCTGACCCTGAAGTTCATCTGCACCACCGGCAAGCTGCCCGTG<br>CCCTGGCCCACCCTCGTGACCACCCTGACCTACGGCGTGCAGTGCTTCAGCCGCTACC<br>CCGACCACATGAAGCAGCACGACTTCTTCAAGTCCGCCATGCCCGAAGGCTACGTCC<br>AGGAGCGCACCATCTTCTTCAAGGACGACGGCAACTACAAGACCCGCGCCGAGGTG<br>AAGTTCGAGGGCGACACCCTGGTGAACCGCATCGAGCTGAAGGGCATCGACTTCAA<br>GGAGGACGGCAACATCCTGGGGCACAAGCTGGAGTACAACTACAACAGCCACAACG<br>TCTATATCATGGCCGACAAGCAGAAGAACGGCATCAAGGTGAACTTCAAGATCCGCC<br>ACAACATCGAGGACGGCAGCGTGCAGCTCGCCGACCACTACCAGCAGAACACCCCC<br>ATCGGCGACGGCCCCGTGCTGCTGCCCGACAACCACTACCTGAGCACCCAGTCCAAA<br>CTGAGCAAAGACCCCAACGAGAAGCGCGATCACATGGTCCTGCTGGAGTTCGTGACC<br>GCCGCCGGGATCACTCTCGGCATGGACGAGCTGTACAAGGGATCCGCAGAAATCGGT<br>ACTGGCTTTCCATTCGACCCCCATTATGTGGAAGTCCTGGGCGAGCGCATGCACTACG<br>TCGATGTTGGTCCGCGCGATGGCACCCCTGTGCTGTTCCTGCACGGTAACCCGACCTC<br>CTCCTACGTGTGGCGCAACATCATCCCGCATGTTGCACCGACCCATCGCTGCATTGCTC<br>CAGACCTGATCGGTATGGGCAAATCCGACAAACCAGACCTGGGTTATTTCTTCGACGA<br>CCACGTCCGCTTCATGGATGCCTTCATCGAAGCCCTGGGTCTGGAAGAGGTCGTCCTG<br>GTCATTCACGACTGGGGCTCCGCTCTGGGTTTCCACTGGGCCAAGCGCAATCCAGAG<br>CGCGTCAAAGGTATTGCATTTATGGAGTTCATCCGCCCTATCCCGACCTGGGACGAAT<br>GGCCAGAATTTGCCCGCGAGACCTTCCAGGCCTTCCGCACCACCGACGTCGGCCGCA<br>AGCTGATCATCGATCAGAACGTTTTTATCGAGGGTACGCTGCCGATGGGTGTCGTCCG<br>CCCGCTGACTGAAGTCGAGATGGACCATTACCGCGAGCCGTTCCTGAATCCTGTTGAC<br>CGCGAGCCACTGTGGCGCTTCCCAAACGAGCTGCCAATCGCCGGTGAGCCAGCGAA<br>CATCGTCGCGCTGGTCGAAGAATACATGGACTGGCTGCACCAGTCCCCTGTCCCGAA<br>GCTGCTGTTCTGGGGCACCCCAGGCGTTCTGATCCCACCGGCCGAAGCCGCTCGCCT<br>GGCCAAAAGCCTGCCTAACTGCAAGGCTGTGGACATCGGCCCGGGTCTGAATCTGCT<br>GCAAGAAGACAACCCGGACCTGATCGGCAGCGAGATCGCGCGCTGGCTGTCGACGC<br>TGGAGATTTCCGGCACCGGTTTGGCGGAGGCGGCGGCGAAGGAGGCGGCGGCGA<br>AGGAGGCGGCGGCGAAGGAGGCGGCGGCGAAGGCGGCGGCGCATATGGACAAAG<br>ACTGCGAAATGAAGCGCACCACCCTGGATAGCCCTCTGGGCAAGCTGGAACTGTCTG<br>GGTGCGAACAGGGCCTGCACCGTATCATCTTCCTGGGCAAAGGAACATCTGCCGCCG<br>ACGCCGTGGAAGTGCCTGCCCCAGCCGCCGTGCTGGGCGGACCAGAGCCACTGATG<br>CAGGCCACCGCCTGGCTCAACGCCTACTTTCACCAGCCTGAGGCCATCGAGGAGTTC<br>CCTGTGCCAGCCCTGCACCACCCAGTGTTCCAGCAGGAGAGCTTTACCCGCCAGGTG<br>CTGTGGAAACTGCTGAAAGTGGTGAAGTTCGGAGAGGTCATCAGCTACAGCCACCTG<br>GCCGCCCTGGCCGGCAATCCCGCCGCCACCGCCGCCGTGAAAACCGCCCTGAGCGG<br>AAATCCCGTGCCCATTCTGATCCCCTGCCACCGGGTGGTGCAGGGCGACCTGGACGT<br>GGGGGGCTACGAGGGCGGGCTCGCCGTGAAAGAGTGGCTGCTGGCCCACGAGGGC<br>CACAGACTGGGCAAGCCTGGGCTG GGTCAATTGCACCATCACCATCACCAC

Supplementary Information

**LynG**	ATGGGATGTATCAAGAGTAAGCGTAAGGATAATCTCAATGACGACGAGCTCGAGACC<br>ATGGTGAGCAAGGGCGAGGAGCTGTTCACCGGGGTGGTGCCCATCCTGGTCGAGCT<br>GGACGGCGACGTAAACGGCCACAAGTTCAGCGTGTCCGGCGAGGGCGAGGGCGAT<br>GCCACCTACGGCAAGCTGACCCTGAAGTTCATCTGCACCACCGGCAAGCTGCCCGTG<br>CCCTGGCCCACCCTCGTGACCACCCTGACCTACGGCGTGCAGTGCTTCAGCCGCTACC<br>CCGACCACATGAAGCAGCACGACTTCTTCAAGTCCGCCATGCCCGAAGGCTACGTCC<br>AGGAGCGCACCATCTTCTTCAAGGACGACGGCAACTACAAGACCCGCGCCGAGGTG<br>AAGTTCGAGGGCGACACCCTGGTGAACCGCATCGAGCTGAAGGGCATCGACTTCAA<br>GGAGGACGGCAACATCCTGGGGCACAAGCTGGAGTACAACTACAACAGCCACAACG<br>TCTATATCATGGCCGACAAGCAGAAGAACGGCATCAAGGTGAACTTCAAGATCCGCC<br>ACAACATCGAGGACGGCAGCGTGCAGCTCGCCGACCACTACCAGCAGAACACCCCC<br>ATCGGCGACGGCCCCGTGCTGCTGCCCGACAACCACTACCTGAGCACCCAGTCCAAA<br>CTGAGCAAAGACCCCAACGAGAAGCGCGATCACATGGTCCTGCTGGAGTTCGTGACC<br>GCCGCCGG GATCACTCTCGGCATGGACGAGCTGTACAAG
