# Supplementary material for: ProDOL: a general method to determine the degree of labeling for staining optimization and molecular counting
Source: Nat Methods. 2024 Aug 8;21(9):1708–15. doi: 10.1038/s41592-024-02376-6 (PMC11399104; doi:10.1038/s41592-024-02376-6)
Supplement: Supplementary file 2 — Reporting Summary [file 41592_2024_2376_MOESM2_ESM.pdf]

Corresponding author(s): Dirk-Peter Herten
Last updated by author(s): Apr 30, 2024

Reporting Summary

Nature Portfolio wishes to improve the reproducibility of the work that we publish. This form provides structure for consistency and transparency in reporting. For further information on Nature Portfolio policies, see our Editorial Policies and the Editorial Policy Checklist.

Statistics

For all statistical analyses, confirm that the following items are present in the figure legend, table legend, main text, or Methods section.

n/a	Confirmed	
☐	☒	The exact sample size (n) for each experimental group/condition, given as a discrete number and unit of measurement
☐	☒	A statement on whether measurements were taken from distinct samples or whether the same sample was measured repeatedly
☐	☒	The statistical test(s) used AND whether they are one- or two-sided<br>*Only common tests should be described solely by name; describe more complex techniques in the Methods section.*
☐	☒	A description of all covariates tested
☐	☒	A description of any assumptions or corrections, such as tests of normality and adjustment for multiple comparisons
☐	☒	A full description of the statistical parameters including central tendency (e.g. means) or other basic estimates (e.g. regression coefficient) AND variation (e.g. standard deviation) or associated estimates of uncertainty (e.g. confidence intervals)
☐	☒	For null hypothesis testing, the test statistic (e.g. F, t, r) with confidence intervals, effect sizes, degrees of freedom and P value noted<br>*Give P values as exact values whenever suitable.*
☒	☐	For Bayesian analysis, information on the choice of priors and Markov chain Monte Carlo settings
☒	☐	For hierarchical and complex designs, identification of the appropriate level for tests and full reporting of outcomes
☒	☐	Estimates of effect sizes (e.g. Cohen's d, Pearson's r), indicating how they were calculated

Our web collection on statistics for biologists contains articles on many of the points above.

Software and code

Policy information about availability of computer code

Data collection	MicroManager 1.4.22<br>MicroManager 2.0.1<br>SymPhoTime 64 2.7
Data analysis	ProDOL analysis pipeline (https://github.com/hertenlab/ProDOL)<br>GraphPad Prism v9.5.0 (730)<br>MATLAB v9.11.0<br>Rstudio v1.3.959 with R version 4.0.2<br>SMAP (https://github.com/jries/SMAP)<br>Fiji (https://imagej.net/software/fiji/downloads) version 1.54<br>thunderSTORM (https://zitmen.github.io/thunderstorm/) version 1.3<br>quickPBSA (https://github.com/JohnDieSchere/quickpbsa) version 2021.0.1<br>TestSTORM (https://titan.physx.u-szeged.hu/~adoptim/?page_id=183) version 2.0<br>pycops (unreleased code)

For manuscripts utilizing custom algorithms or software that are central to the research but not yet described in published literature, software must be made available to editors and reviewers. We strongly encourage code deposition in a community repository (e.g. GitHub). See the Nature Portfolio guidelines for submitting code & software for further information.

Data

Policy information about availability of data

All manuscripts must include a data availability statement. This statement should provide the following information, where applicable:

- Accession codes, unique identifiers, or web links for publicly available datasets
- A description of any restrictions on data availability
- For clinical datasets or third party data, please ensure that the statement adheres to our policy

The authors declare that the data supporting the findings of this study are available within the paper and its Supplementary Information files. Should any raw data files be needed in another format they are available from the corresponding author upon reasonable request.

Human research participants

Policy information about studies involving human research participants and Sex and Gender in Research.

Reporting on sex and gender	N/A
Population characteristics	N/A
Recruitment	N/A
Ethics oversight	N/A

Note that full information on the approval of the study protocol must also be provided in the manuscript.

Field-specific reporting

Please select the one below that is the best fit for your research. If you are not sure, read the appropriate sections before making your selection.

☒ Life sciences ☐ Behavioural & social sciences ☐ Ecological, evolutionary & environmental sciences

For a reference copy of the document with all sections, see nature.com/documents/nr-reporting-summary-flat.pdf

Life sciences study design

All studies must disclose on these points even when the disclosure is negative.

Sample size	No statistical methods to determine sample sizes were used. Unless stated otherwise, sample number refers to the number of cells analyzed per condition.
Data exclusions	Data was acquired by automated microscopy and cells lacking specific eGFP expression were excluded after acquisition and before DOL analysis. Additionally, the ProDOL software includes a visual inspection software to remove cells where segmentation was not successful. Other data was not excluded unless stated otherwise in the text.
Replication	Data was obtained from multiple experiments as detailed in the manuscript and the supplemental information. Reported results could be replicated across multiple experiments with replicates generating similar results.
Randomization	No randomization was used in the experiments.
Blinding	Blinding was not possible due to the nature of the study.

Reporting for specific materials, systems and methods

We require information from authors about some types of materials, experimental systems and methods used in many studies. Here, indicate whether each material, system or method listed is relevant to your study. If you are not sure if a list item applies to your research, read the appropriate section before selecting a response.

Materials & experimental systems

n/a	Involved in the study
☐	☒ Antibodies
☐	☒ Eukaryotic cell lines
☒	☐ Palaeontology and archaeology
☒	☐ Animals and other organisms
☒	☐ Clinical data
☒	☐ Dual use research of concern

Methods

n/a	Involved in the study
☒	☐ ChIP-seq
☒	☐ Flow cytometry
☒	☐ MRI-based neuroimaging

Antibodies

Antibodies used

1. α-SLP76-PY145 (Rabbit) - monoclonal IF - Abcam (ab206782) - clone EP2853Y - 2 µg/ml
2. α-GFP (Mouse) - polyclonal WB - Cell Biolabs (part 212101) - n/a -1:1000 dilution
3. α-CD3 (Mouse) - monoclonal surface preparation - BioLegend (V CD03.05) -clone HIT3a - 1:50 dilution at 10 µg/ml
4. α-mouse-IgG conjugated with HRP (Goat) - polyclonal WB - Jackson Immuno- Research (115-035-003) - n/a - 1:10000 dilution

Validation

All antibodies were commercially available and validated by the manufacturer. Furthermore, they have been used in previous publication and staining with all antibodies yielded the expected spatial distribution/localisation of the protein of interest.
1. The antibody has been validated for FlowCyt, WB, Dot blot and IF by the manufacturer.
2. Validated by ELISA by the manufacturer.
3.FC -Quality tested. Activ -Reported in the literature:
Sedelies KA, et al. 2004. J. Biol. Chem. 279:26581. (Activ)
Rivollier A, et al. 2004. Blood 104:4029. (Activ)
Scharschmidt E, et al. 2004. Mol. Cell Biol. 24:3860. (Activ)
Smeltz RB. 2007. J. Immunol. 178:4786. (Activ)
4. Based on immunoelectrophoresis and/or ELISA, the antibody reacts with whole molecule mouse IgG. It also reacts with the light chains of other mouse immunoglobulins. No antibody was detected against non-immunoglobulin serum proteins. The antibody may cross-react with immunoglobulins from other species as reported by the manufacturer.

Eukaryotic cell lines

Policy information about cell lines and Sex and Gender in Research

Cell line source(s)

1. H838 (NCI-H838, ATCC)
2. HeLa (CCL-2,ATCC)
3. HEK293T were obtained from the laboratory of Ralf Bartenschlager (Heidelberg University)
4. Jurkat cells were obtained from the laboratory of Oliver Fackler (University Hospital Heidelberg)
5. U2OS cells were obtained from the laboratory of Jan Ellenberg (EMBL Heidelberg).
6. Huh-7.5 cells were obtained from the laboratory of Ralf Bartenschlager (Heidelberg University)

Authentication

1. Cell line authentication was performed using Multiplex Cell Authentication by Multiplexion (Heidelberg, Germany) as described (Castro et al, 2013).
2. Cell line authentication was performed using Multiplex Cell Authentication by Multiplexion (Heidelberg, Germany) as described (Castro et al, 2013).
3. Cell line was not authenticated
4. Cell line was not authenticated
5. Authenticated via STR profiling by manufacturer
6. Cell line authentication was performed using Multiplex Cell Authentication by Multiplexion (Heidelberg, Germany) as described (Castro et al, 2013).

Castro F, Dirks WG, Fahnrich S, Hotz-Wagenblatt A, Pawlita M, Schmitt M (2013) High-throughput SNP-based authentication of human cell lines. Int J Cancer 132: 308 – 314

Mycoplasma contamination

Cell lines have been regularly tested for mycoplasma contamination with negative results.

Commonly misidentified lines
(See ICLAC register)

No commonly misidentified cell lines were used in the study.
