# Supplementary material for: ProDOL: a general method to determine the degree of labeling for staining optimization and molecular counting
Source: Nat Methods. 2024 Aug 8;21(9):1708–15. doi: 10.1038/s41592-024-02376-6 (PMC11399104; doi:10.1038/s41592-024-02376-6)
Supplement: Supplementary file 4 — Description of the replicate numbers for all relevant figures. [file 41592_2024_2376_MOESM4_ESM.pdf]

Fig 2 (n in cells)	ELE	quickPBSA	CoPS	ProDOL
f	16	15	11	29
g	-	-	11	17

Fig. 3. a) I) and III) (n in cells)								
concentration (nM)	0	0.1	1	5	10	50	100	250
pre-fixation	17	10	14	17	14	15	12	12
post-fixation	6	9	14	11	10	10	11	11

Fig. 3 a) II) and IV) (n in cells)								
concentration (nM)	0	0.1	1	5	10	50	100	250
pre-fixation	18	20	18	16	17	18	16	17
post-fixation	18	18	0	13	13	17	15	16

Fig. 3 b) (n in cells)						
concentration (nM)		15min	30min	1h	3h	16h
	0.1	30	31	34	33	30
	1	30	33	32	32	50
	5	30	35	32	37	31
	10	30	32	33	35	38
	50	30	34	34	34	33
	100	30	33	33	31	34

Fig. 4 a)

ProDOL (n in cells)	8%	12%	20%
	21	20	9

CoPS (n in clusters)	1.5 min	5 min	10 min
8% DOL	110	268	72
12% DOL	59	175	41
20% DOL	65	237	41

Fig. 4 b)			
ProDOL (n in cells)	16.70%		
	24		
CoPS (n in clusters)	eGFP	eGFP-Nef	
	1348	380	
Extended Data Fig. 2 (images)	20		
Extended Data Fig. 3 (images)	20		
Extended Data Fig. 4 (cells)	ProDOL	LynG	WT
	194	189	38
Extended Data Fig. 5 (clusters)	ELE	PBSA	CoPS
	2352	3668	1626

Extended Data Fig. 6 (n in cells)

	live JF549 1 nM	live JF549 10 nM	live SiR 1 nM	live SiR 10 nM	fixed SiR 0.1 nM	fixed AF647 0.1 nM	fixed AF647 1 nM	live JF646 1 nM	live JF646 10 nM
a) HaloTag DOL	20	16	20	18	42	34	40	17	18

	live JF549 5 nM	live JF549 100 nM	live TMR 5 nM	live TMR 100 nM	live JF646 5 nM	live JF646 100 nM	fixed control	fixed AF647 0.1 nM	fixed AF647 1 nM
b) SNAP-tag DOL	15	19	19	19	18	16	33	38	35

	live control	live JF549 10 nM	live control	fixed control	live SiR 10 nM	fixed SiR 0.1 nM	fixed AF647 0.1 nM	fixed AF647 1 nM	live JF646 10 nM
c) HaloTag unpecific labelling	18	17	20	38	20	33	38	34	19

	live control	live JF549 100 nM	live TMR 100 nM	live control	live JF646 100 nM	fixed control	fixed AF647 0.1 nM	fixed AF647 1 nM
d) SNAP-Tag unspecific labelling	20	19	19	17	18	38	34	29

Extended Data Fig. 7 a) (n in cells)

concentration (nM)	15min	30min	1h	3h	16h
0.001	5	9	6	6	6
0.1	9	8	7	10	5
1	8	8	8	6	10
5	9	9	9	9	8
10	7	7	10	8	7
50	8	7	9	9	10
100	8	9	10	8	9
250	8	10	10	9	7

Extended Data Fig. 7 b) (n in cells)

concentration (nM)	15min	30min	1h	3h	16h
0.01	5	9	6	6	6
0.5	9	8	7	10	5
5	8	8	8	6	10
25	9	9	9	9	8
50	7	7	10	8	7
250	8	7	9	9	10
500	8	9	10	8	9
1250	8	10	10	9	7

Extended Data Fig. 7 c) (n in cells)

concentration (nM)	15min	30min	1h	3h	16h
0.001	8	10	7	7	8
0.1	10	8	10	7	9
1	7	9	9	8	8
5	9	10	9	8	9
10	7	8	7	7	9
50	9	8	8	9	8
100	5	9	9	9	7
250	5	10	10	9	7

Extended Data Fig. 7 d) (n in cells)

concentration (nM)	15min	30min	1h	3h	16h
0.01	8	10	7	7	8
0.5	10	8	10	7	9
5	7	9	9	8	8
25	9	10	9	8	9
50	7	8	7	7	9
250	9	8	8	9	8
500	5	9	9	9	7
1250	5	10	10	9	7

Extended Data Fig. 8 a) (n in cells)

concentration (nM)	15min	30min	1h	3h	16h
0.001	15	13	10	8	12
0.1	7	11	12	13	10
1	7	9	11	10	11
5	11	13	9	14	12
10	8	12	5	12	13
50	10	12	4	9	13
100	14	12	5	9	13
250	11	13	7	14	13

Extended Data Fig. 8 b) (n in cells)

concentration (nM)	15min	30min	1h	3h	16h
0.01	15	13	10	8	12
0.5	7	11	12	13	10
5	7	9	11	10	11
25	11	13	9	14	12
50	8	12	5	12	13
250	10	12	4	9	13
500	14	12	5	9	13
1250	11	13	7	14	13

Extended Data Fig. 8 c) (n in cells)

concentration (nM)	15min	30min	1h	3h	16h
0.001	7	11	7	8	13
0.1	11	8	11	9	10
1	11	6	9	11	9
5	11	10	5	15	8
10	11	14	8	11	12
50	10	14	3	5	9
100	14	10	5	5	12
250	7	12	7	7	14

Extended Data Fig. 8 d) (n in cells)

concentration (nM)	15min	30min	1h	3h	16h
0.01	7	11	7	8	13
0.5	11	8	11	9	10
5	11	6	9	11	9
25	11	10	5	15	8
50	11	14	8	11	12
250	10	14	3	5	9
500	14	10	5	5	12
1250	7	12	7	7	14

Extended Data Fig. 9 (n in cells)

concentration (nM)	15min	30min	1h	3h	16h
0.1	28	25	30	33	31
1	40	45	30	32	30
5	30	30	30	35	30
10	29	30	30	35	30
50	30	30	30	31	30
100	30	30	30	36	30

Supplementary Fig. 2 (n in clusters)	1.5 min	5 min	10 min	eGFP	Nef-eGFP
	800	1211	1206	822	358

Supplementary Fig. 3 b. (n in antibodies)	233

Supplementary Fig. 3 c. (n in cells)	GFP	Nef-GFP
	10	12
